# Parental burnout and adolescents’ academic burnout: Roles of parental harsh discipline, psychological distress, and gender

**DOI:** 10.3389/fpsyg.2023.1122986

**Published:** 2023-02-23

**Authors:** Han Zhang, Shujun Li, Ruimei Wang, Qing Hu

**Affiliations:** ^1^School of Education, Linyi University, Linyi, China; ^2^Department of Education and Training, Weifang People’s Hospital, Weifang, China

**Keywords:** parenting emotion, academic performance, social learning theory, junior high school student, multiple mediation model

## Abstract

Parental burnout is an emerging hot issue in discussions about children’s mental health and development. However, little is known about the underlying psychological mechanisms of parental burnout on children’s academic burnout. To fill in this gap, we aim to examine the relationship between parental burnout and adolescents’ academic burnout, as well as the mediating effects of harsh discipline, adolescents’ psychological distress, and the moderating effect of gender. A sample of 871 junior high school students (477 boys and 394 girls) and their primary caregivers from Eastern China participated in this study. The results showed a direct relationship between parental burnout and academic burnout as well as an indirect relationship through the mediating role of psychological distress and the chain-mediating roles of parental harsh discipline and psychological distress. Furthermore, we discovered that fathers’ parental burnout had a stronger effect on children’s psychological distress than mothers. These findings contribute to our understanding of how parental burnout relates to children’s academic burnout and underline the significance of fathers’ parental burnout.

## 1. Introduction

With the aggravation of the contraction between high expectations and limited energy in parenting, parental burnout gradually rises to a serious social issue in the current era. Parental burnout is defined as a series of negative symptoms brought on by parental role and long-term parenting stress (Mikolajczak et al., 2018b). It not only damages their mental health and behavior, but its negative spillover effect also spread to their spouses and children (Mikolajczak et al., 2018a). Prior research regarding the influence of parental burnout on children tended to focus on parenting practices (such as neglect and violence) and children’s mental health (Mikolajczak et al., 2018a; Mikolajczak et al., 2019; Yang et al., 2021). The influence of parental burnout on academic burnout has received little attention. However, driven by the result-oriented educational background, adolescents’ primary task is to study, and academic burnout is a common issue. Academic burnout plays an important role in adolescents’ growth and social development (Lay-Khim and Bit-Lian, 2019; Ogbueghu et al., 2019). Severe academic burnout leads to a series of maladaptive and negative developmental outcomes, such as poor academic performance, truancy, school dropouts, as well as psychological disorders (Rudolph et al., 2001; Bilige and Gan, 2020). Therefore, understanding the influence of parental burnout on children’s academic performance is crucial to fully understanding its influence on children. It is equally essential to pay attention to parental burnout to reduce adolescents’ academic burnout. The present study aims to examine the relationship between parental burnout and adolescents’ academic burnout, as well as the mediating effects of harsh discipline, adolescents’ psychological distress, and the moderating effect of gender.

### 1.1. Parental burnout and academic burnout

As the demand-resource model of burnout indicates, burnout occurs when an individual’s resources cannot satisfy the demand (Demerouti et al., 2001). In the process of parenting, parental burnout occurs when a parent’s resources are insufficient to meet the demands of parenting (Mikolajczak and Roskam, 2018). Recently, due to the rising prevalence of parental burnout among parents worldwide, researchers have been more interested in this subject. According to a survey conducted in 42 countries throughout the world, the incidence of parental burnout ranged from 0 to 8% (Roskam et al., 2021). Parental burnout has a profound effect on adolescents due to their deficiencies in emotion regulation abilities. Therefore, parental burnout and its effect require exceptional attention. According to social learning theory (Bandura, 1977), children learn and imitate their parents’ behavior patterns and attitudes. This theory has also been confirmed in many research topics, such as addictive behavior and psychological status (Kendler and Gardner, 2017; Xie et al., 2019). Academic burnout refers to a certain type of reaction caused by students’ failure to cope with academic stress (Maslach and Jackson, 1981). Prior researches have also shown that parental burnout has an impact on children’s academic achievement (An et al., 2022) and academic burnout (Wu et al., 2022), but its underlying psychological process was unclear. Therefore, we aim to first examine the relationship between parental burnout and adolescents’ academic burnout, and then analyze its influential mechanism.

### 1.2. The role of parental harsh discipline

According to the theoretical framework of parental burnout, the influence of parental burnout on children’s developmental outcomes may have to be mediated by other factors such as parenting style or parenting behavior (Mikolajczak et al., 2018a). In this way, harsh discipline seems to be an important mediating variable. From the biological point of view, stress elicits and fuels anger (Moons et al., 2010), while emotional and physical exhaustion might limit the executive resources available to suppress negative behaviors (Krabbe et al., 2017). Parental burnout resulted not only in neglect and evasion but also in the violent treatment of children and the appearance of rough parenting behavior (Hansotte et al., 2021). As a type of rough parenting behavior, parental harsh discipline is a common parenting style globally, especially in Chinese families (Wang and Liu, 2014). It refers to the compulsory behavior or negative emotional expression committed by parents against children’s improper behavior (Erath et al., 2009). Burned-out parents are frequently trapped in parenting stress, which might impair their ability to control the occurrence of negative parenting behaviors (Roskam et al., 2017). Thus, burned-out parents are more inclined to adopt harsh discipline to deal with issues that arise during the child-rearing process.

Previous researches on the association between parental harsh discipline and children’s academic performance have reached conflicting conclusions. Parental harsh discipline is frequently interpreted as responsibility and care for children in traditional Chinese culture (Chao, 1994). Furthermore, the examples of “Tiger Mom” and “Wolf Dad” indeed provide evidence for the promotion effect of parental harsh discipline on children’s academic achievement (Chu and Xie, 2023). However, ample studies in recent years have criticized that parental harsh discipline impeded children’s cognitive and emotional development, and increased the degree of social maladjustment (Gershoff et al., 2010; Bai L. et al., 2020). Another study directly linked negative parenting behavior (e.g., punishment) to the risk of academic burnout in children (Luo et al., 2016). As parental acceptance-rejection indicated, neglect and aggression increase children’s risk for problematic behaviors and academic burnout (Rohner, 2004). Accordingly, this study attempt to examine the mediating role of parental harsh discipline between parental burnout and adolescents’ academic burnout.

### 1.3. The role of psychological distress

Academic burnout is influenced not only by family factors but also by individual factors (Ling et al., 2014). According to the intergenerational integration model of depression (Goodman and Gotlib, 1999), caregivers who are depressed as a result of parental burnout might create stressful living environments through their negative cognition, emotions, and behaviors, which cause children to internalizing problematic behavior. The empirical studies have also shown that parental burnout harmed children’s mental health (Yang et al., 2021). However, different from mental health, psychological distress is characterized as an unpleasant emotional state that individuals experience when they respond to the demands that cause psychological disorders (Ridner, 2004). Although existing evidence has supported the link between parental burnout and mental health, it was indefinite whether similar relations existed between parental burnout and psychological distress. Clarifying their connections is beneficial to enrich the knowledge about the influence of parental burnout on children’s psychological situations.

Prior studies have investigated the association between psychological distress and academic burnout but reached the opposite conclusion. Smith and Emerson (2021) have indicated that academic burnout and its dimensions had effects on psychological distress, whereas other scholars thought that negative emotions worsened academic burnout (Salmela-Aro et al., 2009; Zhang et al., 2022). Existing research, however, has primarily focused on college students, and this relationship among junior high school students was not well explored. The theory of resource conservation holds that individual resources are limited (Ford, 2007). If an individual must spend resources on psychological adjustment, the resources available for learning will be limited, contributing to higher academic burnout. We tend to regard psychological distress as an antecedent of academic burnout. According to the theory of the mediation effect proposed by Baron and Kenny (1986), psychological distress is likely to play mediating role in the relationship between parental burnout and academic burnout. Therefore, examining the mediating role of psychological distress is also our aim.

### 1.4. The relations between parental harsh discipline and psychological distress

Since both parental harsh discipline and psychological distress might mediate the relationship between parental burnout and academic burnout, is there any link between these two mediators? Although no direct evidence existed to support the association between parental harsh discipline and the psychological distress of children, it has been confirmed in related concepts. As the emotional security proposition argues, parental harsh discipline might weaken individual emotional security, leading to anxiety, anger, and other feelings in children (Cummings and Davies, 1996). A prior study has shown that parental harsh discipline drastically increased the risk of internalizing problematic problems (e.g., depression and anxiety; Wang et al., 2016). Therefore, we speculate that parental harsh discipline is positively related to children’s psychological distress, and that the two variables play chain-mediating effects in the relations between parental burnout and academic burnout.

### 1.5. Gender differences in the mediating mechanism

Previous studies have found that parental harsh discipline and children’s internalizing and externalizing problematic behavior may vary based on gender (Chang et al., 2003; Liu et al., 2022). According to the social role theory, society has distinct expectations on different genders, which are what make up gender roles (Eagly et al., 2000). The expectations and cognitions of parents’ gender roles could affect children’s perception and interpretation of their behaviors. Therefore, the same behavior may have a very degree of effect on children due to gender differences. Father’s harsh discipline has a stronger effect on boys’ aggressive behavior than it does on girls, whereas a mother’s harsh discipline does not affect both boys and girls (Chang et al., 2003). Relevant research also showed gender consistency in the relationship between parental harsh discipline and children’s anxiety (Deater-Deckard and Dodge, 1997). Based on these evidences, this study aims to investigate whether there are gender differences in this sequential mediation model of parental burnout and adolescents’ academic burnout. The research results are helpful to increase the knowledge of gender differences in the relationship between parental burnout and children’s development.

### 1.6. The present study

**Figure 1:**
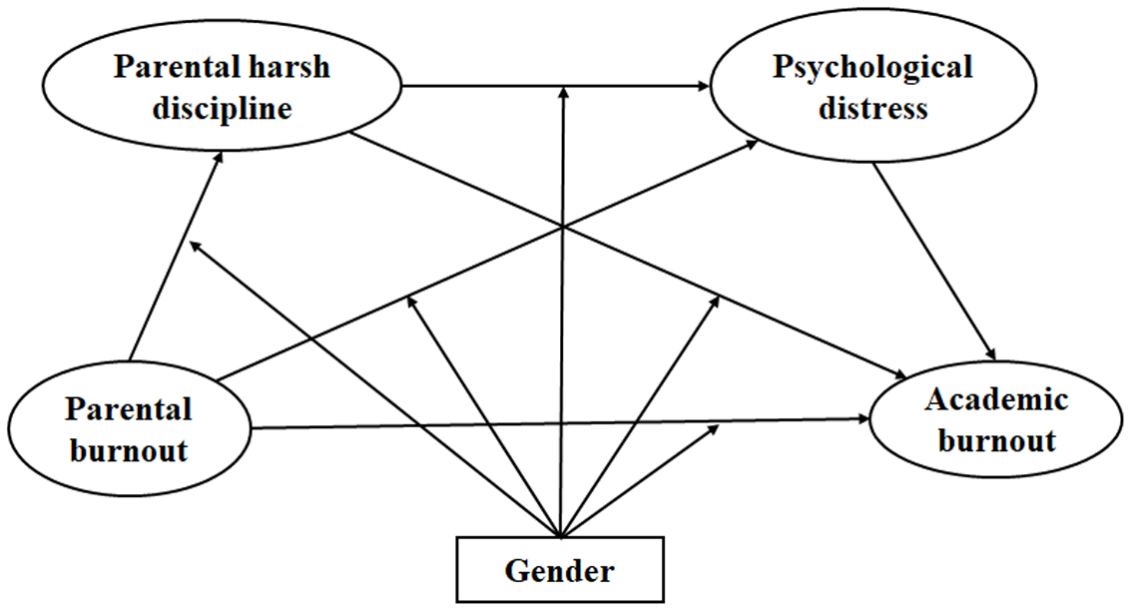
The theoretical model.

To make up for the deficiency in the literature, the present study aims to examine the relationship between parental burnout and adolescents’ academic burnout, as well as the mediating roles of harsh discipline, adolescents’ psychological distress, and the moderating role of gender. To solve this problem, we conducted a questionnaire survey on adolescents and their parents and provided evidence for this study from a multi-subject perspective. Based on relevant theories and previous empirical studies, we proposed the following hypotheses. (1) Parental burnout is positively related to children’s academic burnout. (2) Parental harsh discipline mediates the relations between parental burnout and academic burnout. (3) Children’s psychological distress also plays a mediating role in the association between parental burnout and academic burnout. (4) Parental harsh discipline and psychological distress play chain-mediating roles between parental burnout and academic burnout. (5) The mediating mechanism has gender differences and the mediation effect is more significant in mothers than in fathers. The detailed theoretical model was shown in Figure 1.

## 2. Method

### 2.1. Participants and procedures

We conducted this study in the Shandong Province of China during the first week of May 2022. Since offline questionnaires were unavailable during the COVID-19 pandemic, and web surveys had a similar effect to offline surveys (Huang, 2006), we carried out this investigation through a web survey tool named Wenjuanxing. Considering the difference in lockdown time, we just recruited students of Grade 7 and Grade 8 to participate in this survey. Those students and their primary caregivers (only father or mother) who voluntarily participated, were included in this study. Yet, students and their parents who suffered or were suffering psychological, neurological, or major physiological diseases were excluded. This study was approved by the ethics committee of the first author’s institution. Informed content was obtained from all persons, only those who clicked on the agree option could answer the latter questionnaires. All persons were free to withdraw from this survey at any time and without any reward or punishment.

The survey was divided into two parts: a questionnaire for parents and a questionnaire for students. The parent questionnaire was mainly used to collect demographic characteristics of parents and family-related information, parental burnout, and parental harsh discipline. The student questionnaire contained more detailed information, including demographic characteristics of students, academic burnout, and psychological distress. A total of 1,015 students and 1,030 parents completed the questionnaire. After matching the questionnaires of students and their parents using the students’ ID numbers, 871 students and parents whose responses were valid and finally included in this study. The boys and girls were 477 (53.8%) and 394 (45.2%), respectively. Their ages ranged from 11 to 16 years with a mean age of 13.75 (0.74) years old. More than half of the students came from Grade 8, which accounted for 61.2%. Among the valid parents’ questionnaires, the proportion of mothers and fathers was 73.8% (643/871) and 26.2% (228/871). The average age of parents was 39.14 (3.08) years old. All the persons were Han Chinese and native Chinese speakers. Of 595 families (68.3%) had two children.

### 2.2. Measurements

#### 2.2.1. Parental burnout

Parental Burnout Assessment (PBA), developed by Roskam et al. (2018) and translated by Cheng et al. (2020), was used to measure parental burnout. It contains 23 items and consists of four subscales: exhaustion in parental role, contrast in parental self, feelings of being fed up and emotional distancing. Items were responded on a 7-point Likert scale ranging from 1 (i.e., never) to 7 (i.e., everyday). A higher score indicates stronger feelings of parental burnout parents perceived. In the current study, the scale was completed by parents. The Cronbach alpha of the total scale was 0.96.

#### 2.2.2. Parental harsh discipline

Parental harsh discipline was measured using the Parent–child Conflict Tactics Scales (CTSPC), which was compiled by Straus et al. (1998). This scale was widely used to measure parental corporal punishment among adolescents and demonstrated to have high validity and reliability (Wang and Liu, 2018). In the current study, we only used the subscales of psychological aggression and corporal punishment to measure the frequency of harsh discipline by parents in the past half-year. The scale uses a 7-point scoring standard that ranges from 0 to 6, indicating the frequency of harsh discipline. According to the instruction of CTSPC Straus and colleagues provided, we calculated frequency based on the median number of times parents scored on each item (Straus et al., 1998). The higher they scored, the more psychological aggression or corporal punishment their parents imposed on their children. The Cronbach alpha of this scale in the present study was 0.89.

#### 2.2.3. Psychological distress

Kessler 10 was used to estimate the psychological distress of students in junior high school. This scale was developed by Kessler et al. (2002) and translated into Chinese by Huang et al. (2009). It is one of the most widely used self-rated measuring tools. It contains 10 items and the response was rated on a 5-point Likert scale. Persons were asked to rate their psychological condition in past 4 weeks. The total score ranged from 10 to 50, and a higher score indicated more frequent psychological distress. The Cronbach alpha of the scale in this study was 0.95.

#### 2.2.4. Academic burnout

Academic burnout was assessed by the Academic Burnout Scale constructed by Wu et al. (2007) based on Maslach Burnout Inventory. It was a self-rated scale used to measure the academic burnout of adolescents. This scale contains 16 items with three subscales, namely, physical and psychological exhaustion, academic alienation and low sense of accomplishment. Six items were reversely scored and needed to be transformed. The responses were rated on a 5-point scale, with 1 and 5. A higher score represents severer academic burnout. In the current study, the Cronbach alpha of the scale in this study was 0.88.

### 2.3. Data analysis

The data for this study was collected online, and persons were not allowed to skip any questions when answering. Therefore, no data was missing. SPSS 23.0 and AMOS 23.0 software were employed to perform all analyzes. After checking for normality, we log-transformed parental burnout and parental harsh discipline. We still used the original data to clearly present the initial scores of variables but used the transformed data in the other analyzes. The correlations between every two variables were primarily described using correlation analysis. We used the Structural Equation Model (SEM) to further analyze the link between parental burnout, parental harsh discipline, psychological distress and academic burnout. According to the suggestion of Rogers and Schmitt (2004), we adopted item parceling strategies for the unidimensional variable (psychological distress) to control the inflated measurement error. In this process, we first conducted a factor analysis to rank the items according to the value of loadings. After that, arrange items in turn from high to low and vice versa. This method balanced the loading and variance of each package to the greatest extent. The average scores of each package were used as observational variables of psychological distress. Multiple group structural equation model was also used to compare the mediation model in different genders of primary caregivers. The model fit could be considered good if *χ*^2^/df < 3, RMSEA < 0.05, SRMR < 0.05, GFI > 0.90, CFI > 0.90, and NFI > 0.90. We performed the bias-corrected bootstrap to examine the mediating effect based on 5,000 samples. A *p* value lower than 0.05, confidence intervals did not include zero, or critical ratios for differences between parameters higher than 1.96 were considered significant.

## 3. Results

### 3.1. Preliminary analyzes

We first conducted correlation analysis to get a preliminary understanding of the relationship between variables. As expected, all variables were positively correlated, detailed information could be found in Table 1.

**Table 1:** Bivariate correlations (*N* = 871).

Variables	*M*	SD	PB	PHD	PD	AB
Parental burnout (PB)	45.70	24.73	1			
Parental harsh discipline (PHD)	19.87	30.18	0.55^**^	1		
Psychological distress (PD)	18.55	7.89	0.35^**^	0.29^**^	1	
Academic burnout (AB)	38.21	9.48	0.39^**^	0.33^**^	0.59^**^	1

M, mean; SD, standard deviation; ^**^p < 0.01.

### 3.2. Mediation models

To examine the first four research hypotheses, namely the direct and indirect relationship between parental burnout and children’s academic burnout, we have established several structural equation models. When examining the direct relationship between parental burnout and academic burnout, the model fit showed acceptable (*χ*^2^/df = 2.724, RMSEA = 0.045, SRMR = 0.020, GFI = 0.991, CFI = 0.996, NFI = 0.993). Then we constructed a multiple mediation model to deeply analyze the indirect relationships. The results showed that all indicators of model fit were good (*χ*^2^/df = 2.663, RMSEA = 0.044, SRMR = 0.020, GFI = 0.978, CFI = 0.991, NFI = 0.985). Parental burnout was found to be a significant independent variable in academic burnout (*β* = 0.16, *p* < 0.001). It also had a positive effect on parental harsh discipline (*β* = 0.61, *p* < 0.001) and psychological distress (*β* = 0.26, *p* < 0.001), both of which were mediators in the current model. Psychological distress was positively related to academic burnout (*β* = 0.58, *p* < 0.001). However, the other mediator, parental harsh discipline, was not significantly associated with academic burnout (*β* = 0.07, *p* = 0.15). Additionally, parental harsh discipline was linked to a higher risk of psychological distress (*β* = 0.19, *p* < 0.001).

**Figure 2:**
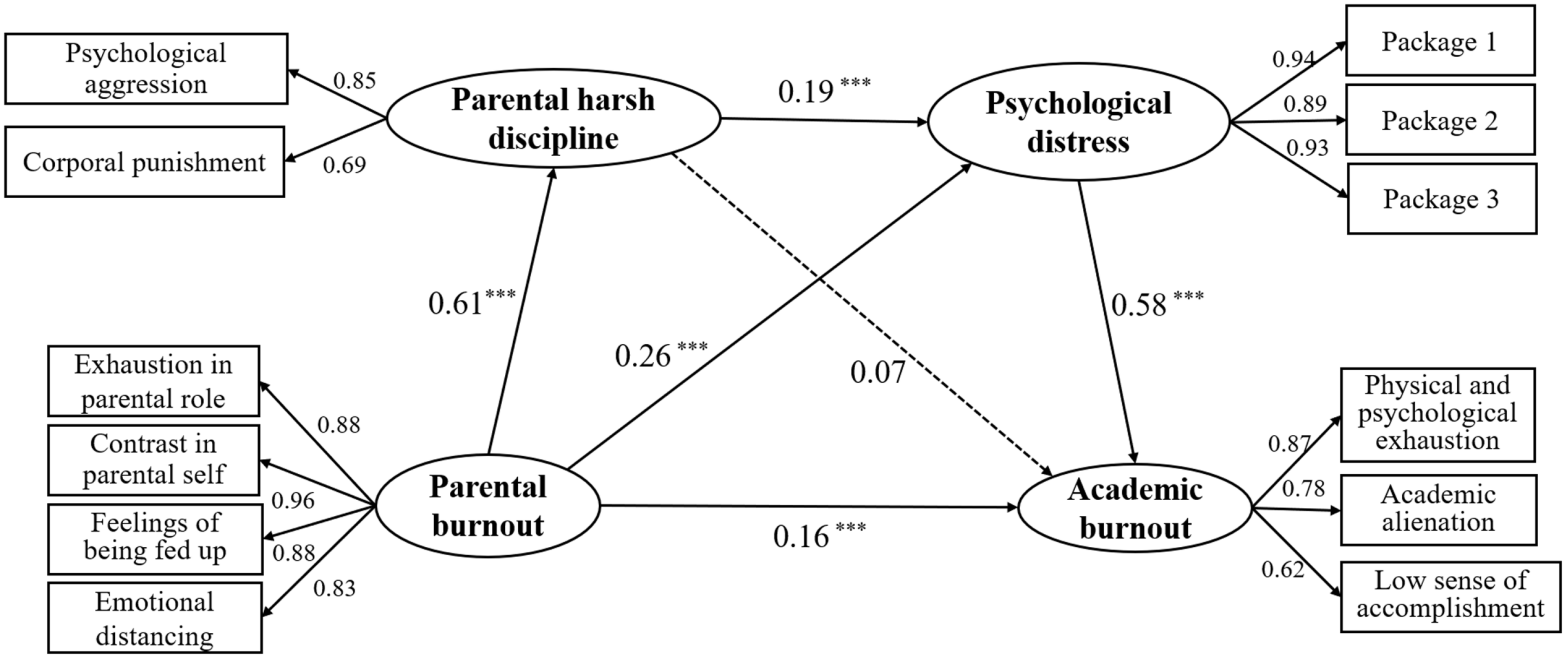
The multiple mediation model. All path coefficients were standardized. ^*^p < 0.05, ^**^p < 0.01, ^***^p < 0.001.

As shown in Figure 2, three indirect paths were found in the model: (1) parental burnout → parental harsh discipline → academic burnout; (2) parental burnout → psychological distress → academic burnout; (3) parental burnout → parental harsh discipline → psychological distress → academic burnout. Relevant results are displayed in Table 2. For the first path, the indirect effect of parental burnout on academic burnout through parental harsh discipline was not significant (*p* = 0.171). For the second path, parental burnout was positively related to academic burnout through the mediating role of psychological distress (*p* < 0.001), and this path coefficient was higher than the other two indirect paths. The last chain-mediating path was also significant (*p* = 0.001), parental burnout had a positive effect on parental harsh discipline and psychological distress, which in turn contributed to academic burnout. In conclusion, parental burnout has a significant indirect effect on academic burnout. Under the given situation, its direct effect also had significance. Parental harsh discipline and psychological distress partially mediated the effect of parental burnout on academic burnout, accounting for 61.2% of the total effect.

**Table 2:** Indirect effects of parental burnout on academic burnout.

Indirect path	Effect value	*P*	Boot LLCI	Boot ULCI
Path 1: PB → PHD → AB	0.040	0.171	−0.017	0.099
Path 2: PB → PD → AB	0.150	<0.001	0.094	0.210
Path 3: PB → PHD → PD → AB	0.067	0.001	0.029	0.107
Total indirect effect	0.257	<0.001	0.188	0.328

PB = Parental burnout; PHD = Parental harsh discipline; PD = Psychological distress; AB = Academic burnout; LLCI = Lower limit confidence interval; ULCI = Upper limit confidence interval.

### 3.3. Multiple group path analysis

We constructed a multiple group structural equation model to examine the fifth hypothesis, namely comparing multiple mediation models in different genders of primary caregivers. Table 3 presents the standardized path coefficients in different groups, as well as critical ratios for parameter differences. The critical ratio for parameter differences between mother and father on the path from parental burnout to adolescents’ psychological distress was 2.29, which was higher than 1.96. Thus, these path coefficients varied significantly between different genders of primary caregivers. Specifically, the path coefficient from parental burnout to psychological distress in mother-cared students (*β* = 0.19, *p* < 0.001) was lower than father-cared students (*β* = 0.47, *p* < 0.001).

**Table 3:** Standardized path coefficients in different gender of primary caregivers.

Path	Primary caregiver (mother/father)		
	*β*	C.R.	C.R. for Differences between Parameters
PB to PHD	0.58/0.70	11.10^***^/8.29^***^	0.66
PB to AB	0.24/0.03	4.15^***^/0.29	−1.58
PB to PD	0.19/0.47	3.72^***^/4.67^***^	2.29
PHD to AB	0.09/−0.07	1.81/−0.66	−1.32
PHD to PD	0.59/0.56	11.82^***^/6.66^***^	0.25
PD to AB	0.15/0.26	3.25^**^/2.55^*^	1.03

C.R. = Critical Ratios; PB = Parental burnout; PHD = Parental harsh discipline; PD = Psychological distress; AB = Academic burnout; *p < 0.05, **p < 0.01, ***p < 0.001.

## 4. Discussion

Family-related factors have a significant impact on adolescents’ academic burnout. However, little is known about the influential mechanism of parental burnout on children’s academic burnout. To fill in this gap, we constructed structural equation model and first examined these relationships. We found that parental burnout had a direct relationship with academic burnout as well as an indirect relationship through the mediating role of psychological distress, and the chain-mediating roles of parental harsh discipline and psychological distress. Furthermore, fathers’ parental burnout had a stronger effect on children’s psychological distress than mothers.

### 4.1. Relations between parental burnout and academic burnout

This study found a positive relationship between parental burnout and academic burnout, which indicated that parenting emotion is a factor in adolescents’ academic burnout. This finding was consistent with a prior study that regarded parental burnout as a mediator and found that parental burnout mediated the relationship between parents’ education anxiety and children’s academic burnout (Wu et al., 2022). Other relevant research also revealed that a favorable upbringing was conducive to obtaining emotional warmth and support from their parents, and that as a result, they were capable of coping with academic stress and showed less academic burnout (He et al., 2017). The results enriched the social learning theory (Bandura, 1977), and extended it to the field of burnout. Therefore, improving parents’ feelings about parenting and letting parents play an exemplary role are crucial to intervene in children’s academic burnout, especially during the COVID-19 lockdown.

### 4.2. The mediating role of parental harsh discipline

Contrary to the hypothesis, although parental burnout was linked to harsh discipline, parental harsh discipline failed to be associated with academic burnout. That is to say, the mediating path did not reach a significant level in the latter half. This result differed from previous studies which have found either positive or negative relations between parental harsh discipline and academic burnout (Gershoff et al., 2010; Chu and Xie, 2023). Several reasons can be used to explain this finding. First, parental harsh discipline may have an indirect effect on academic burnout. Previous studies have found that individual factors had a great influence on academic burnout (Yang and Cheng, 2005; Ling et al., 2014), and harsh discipline affected children’s academic burnout through psychological capital and self-control (He et al., 2017). Second, the inconsistency of the findings resulted from cultural differences. Previous study in the eastern country has found that parental harsh discipline was beneficial for adolescents’ academic achievements (Chu and Xie, 2023). Although the influence of parental harsh discipline on adolescents’ academic burnout is not significant in this study, we still found that mothers’ and fathers’ harsh discipline had opposite effects on academic burnout. Specifically, the father’s harsh discipline is beneficial for children’s academics, while the mother’s harsh discipline is the opposite. It can explain this result to some extent. Third, this study was carried out during the COVID-19 lockdown, the influence of harsh discipline on children’s academic burnout may be offset by other environmental factors or their psychological distress. However, it is worth mentioning that deep and detailed research into their relationship should be carried out.

### 4.3. The mediating role of psychological distress

Aligned with the hypothesis, the mediating model demonstrated that parental burnout had an effect on academic burnout through psychological distress. On the one hand, parental burnout increased children’s psychological distress, which confirmed finding in previous research (Yang et al., 2021). Firstly, burned-out parents provided less social support, and their children were prone to psychological distress (Yang et al., 2021). Burned-out parents were more likely to lose their patience with their children, try to avoid their parenting responsibilities, and gradually ignore their children’s physiological and emotional requirements (Roskam et al., 2017). Secondly, this finding supported the intergenerational integration model of depression (Goodman and Gotlib, 1999). Parental burnout worsened parents’ mental health, generating depressive symptoms and hence psychological distress in their children. On the other hand, as an antecedent variable, psychological distress had an effect on academic burnout, which supported the loss spiral effect of the resource conservation model (Ford, 2007). Psychological distress represents the loss of psychological resources, which puts individuals in a state of stress. This situation resulted in the deficiency of resources and a “chain reaction” of resource loss, which further led to academic burnout. The previous study has also indicated that positive emotions promoted academic engagement and ultimately led to academic success (Durlak et al., 2011). In other words, psychological stress impeded academic engagement and eventually led to academic burnout. The results increased knowledge for understanding the relationship between parental burnout and academic burnout from the perspective of individual psychological distress.

### 4.4. The multiple mediating roles of parental harsh discipline and psychological distress

The present study also discovered that harsh discipline and psychological distress played chain-mediating roles between parental harsh discipline and academic burnout, which was in line with hypothesis four. Parental burnout was associated with harsh discipline and children’s psychological distress, and then linked to academic burnout. The result suggested that although parental harsh discipline cannot mediate the relationship between parental burnout and academic burnout, it can increase psychological distress, and then leads to academic burnout. The effect of parental harsh discipline on children’s psychological distress supported the emotional security hypothesis (Cummings and Davies, 1996). This result can also be explained by the parental acceptance-rejection theory (Rohner, 2004), which suggests that children might misinterpret their parents’ harsh discipline as rejection and thus resulting in psychological distress. This finding was in line with a prior study that showed positive correlations between harsh discipline and psychological distress (Wang et al., 2016). The findings provided a new perspective for the explanation and intervention of academic burnout from the perspective of parental harsh discipline and adolescents’ psychological distress.

### 4.5. Primary caregiver gender differences in the mediating mechanism

Although this study found that the influence of parental burnout on children’s psychological distress differed by caregiver gender, the specific finding is inconsistent with the hypothesis. In comparison to mothers, fathers’ parental burnout had a stronger effect on children’s psychological distress. This result was close to that of a study conducted in French-and English-speaking countries. They discovered that gender moderated the relationship between parental burnout, avoidance of ideas, neglect, and violent behavior toward children (Roskam and Mikolajczak, 2020). The results may be attributed to parenting styles parents adopted after encountering parental burnout. Fathers adopted a more neglectful parenting style, while mothers used severe harsh discipline (Roskam and Mikolajczak, 2020). Parents who employ a neglectful parenting style are constantly disregarding basic demands in children’s development, resulting in their basic psychological demands not being satisfied (Kantor et al., 2004), which brought psychological distress to adolescents (Arslan, 2017). In other words, neglect has a stronger negative effect on children’s mental health than harsh discipline, and this phenomenon is more obvious in Chinese culture. Thus, we should attach importance to the neglectful parenting style toward children. Along with increased father involvement in parenting, we are concerned not only about the effect of mothers’ parental burnout on children, but also the influence of fathers’ parental burnout.

### 4.6. Implications

This study has both theoretical and practical implications. Theoretically, this study focused on the negative impact of the emerging hot topic of parental burnout and determined the relationship between parental burnout and adolescents’ academic burnout. This study integrated social learning theory, intergenerational integration model of depression and resource conservation model, and constructed a model of “parenting emotion–parenting behavior–psychology–academic burnout” to explain how parental burnout increased adolescents’ academic burnout. This provided a new theoretical basis for promoting adolescents’ growth and development. In addition, it also showed that the influence of gender should not be ignored when explaining the influence of parental burnout. Practically, this study provided a new perspective for preventing and intervening in academic burnout in adolescents, and had important practical significance. We should be fully aware of the important role of parental burnout in reducing adolescents’ academic burnout and its negative impact on themselves and their children. In addition, adolescents trying to reduce psychological distress can also effectively prevent the negative impact of parental burnout on adolescents’ academic burnout.

### 4.7. Limitations and future directions

Several limitations should be mentioned in this study. First, the present study was carried out just during the COVID-19 lockdown. As a result, it’s impossible to say “whether the intensity of this relationship after COVID-19 differed from that during the COVID-19 lockdown.” Second, this study was a cross-sectional study, which cannot directly infer causality. According to family system theory, not only parental burnout affects adolescents’ academic burnout, but children’s academic burnout influences parental burnout. Thus, to clarify the causality, longitudinal research is needed in the future. Third, concerning gender differences in the effect of parental burnout, we just analyzed the effect of the father or mother’s parental burnout on children. Nevertheless, collecting mothers’ and fathers’ parental burnout, respectively, could provide more persuasive results on gender differences.

## 5. Conclusion

In conclusion, parental burnout had a direct relationship with academic burnout as well as an indirect relationship through the mediating role of psychological distress, and the chain-mediating roles of parental harsh discipline and psychological distress. Furthermore, fathers’ parental burnout had a stronger effect on children’s psychological distress than mothers. These results add to our understanding of how parental burnout relates to children’s academic burnout and underline the importance of fathers’ parental burnout. Parental burnout is an emerging hot topic worldwide, and this kind of work will help us have a more comprehensive understanding on the influence of parental burnout.

## Data availability statement

The raw data supporting the conclusions of this article will be made available by the authors, without undue reservation.

## Ethics statement

The studies involving human participants were reviewed and approved by the ethics committee of Linyi University. Written informed consent to participate in this study was provided by the participants' legal guardian/next of kin.

## Author contributions

HZ conceived of the study, participated in its design, performed the statistical analysis and drafted the manuscript. SL participated in its design and interpretation of the data and helped to revise the manuscript. RW participated in the interpretation of the data and coordination and collected the data. QH conceived of the study, participated in the design and coordination and collected the data. All authors read and approved the final manuscript.

## Funding

This work was supported by Shandong Social Science Planning Fund Program (grant number 22DJYJ02).

## Conflict of interest

The authors declare that the research was conducted in the absence of any commercial or financial relationships that could be construed as a potential conflict of interest.

## Publisher’s note

All claims expressed in this article are solely those of the authors and do not necessarily represent those of their affiliated organizations, or those of the publisher, the editors and the reviewers. Any product that may be evaluated in this article, or claim that may be made by its manufacturer, is not guaranteed or endorsed by the publisher.

## References

[ref1] An N. H. Hong V. T. P. Thao T. T. P. Thao L. N. Khue N. M. Phuong T. T. T.. (2022). Parental burnout reduces primary students' academic outcomes: a multi-mediator model of mindful parenting and parental behavioral control. Fam. J. 30, 621–629. doi: 10.1177/10664807211052482

[ref2] Arslan G. (2017). Psychological maltreatment, coping strategies, and mental health problems: a brief and effective measure of psychological maltreatment in adolescents. Child Abuse Negl. 68, 96–106. doi: 10.1016/j.chiabu.2017.03.023, PMID: 28427000

[ref4] Bai L. Liu Y. Xiang S. (2020). Associations between parental psychological control and externalizing problems: the roles of need frustration and self-control. J. Child Fam. Stud. 29, 3071–3079. doi: 10.1007/s10826-020-01810-5

[ref5] Bandura A. (1977). Social Learning Theory. Oxford, England: Prentice-Hall.

[ref6] Baron R. M. Kenny D. A. (1986). The moderator-mediator variable distinction in social psychological research: concept strategic and statistical considerations. J. Abnorm. Soc. Psychol. 51, 1173–1182. doi: 10.1037/0022-3514.51.6.1173, PMID: 3806354

[ref7] Bilige S. Gan Y. (2020). Hidden school dropout among adolescents in rural China: individual, parental, peer, and school correlates. Asia Pac. Educ. Res. 29, 213–225. doi: 10.1007/s40299-019-00471-3

[ref8] Chang L. Schwartz D. Dodge K. A. McBride-Chang C. (2003). Harsh parenting in relation to child emotion regulation and aggression. J. Fam. Psychol. 17, 598–606. doi: 10.1037/0893-3200.17.4.598, PMID: 14640808 PMC2754179

[ref9] Chao R. K. (1994). Beyond parental control and authoritarian parenting style: understanding Chinese parenting through the cultural notion of training. Child Dev. 65, 1111–1119. doi: 10.2307/1131308, PMID: 7956468

[ref10] Cheng H. Wang W. Wang S. Li Y. Liu X. Li Y. (2020). Validation of a Chinese version of the parental burnout assessment. Front. Psychol. 11:321. doi: 10.3389/fpsyg.2020.00321, PMID: 32231609 PMC7083175

[ref11] Chu X. Xie R. (2023). Parental harsh discipline and cyberbullying perpetration among Chinese college students: why and when are they related? Deviant Behav. 44, 57–74. doi: 10.1080/01639625.2021.2011480

[ref12] Cummings E. M. Davies P. (1996). Emotional security as a regulatory process in normal development and the development of psychopathology. Dev. Psychopathol. 8, 123–139. doi: 10.1017/S0954579400007008

[ref13] Deater-Deckard K. Dodge K. A. (1997). Externalizing behavior problems and discipline revisited: nonlinear effects and variation by culture, context, and gender. Psychol. Inq. 8, 161–175. doi: 10.1207/s15327965pli0803_1

[ref14] Demerouti E. Bakker A. B. Nachreiner F. Schaufeli W. B. (2001). The job demands-resources model of burnout. J. Appl. Psychol. 86, 499–512. doi: 10.1037/0021-9010.86.3.499 11419809

[ref15] Durlak J. A. Weissberg R. P. Dymnicki A. B. Taylor R. D. Schellinger K. B. (2011). The impact of enhancing Students' social and emotional learning: a meta-analysis of school-based universal interventions. Child Dev. 82, 405–432. doi: 10.1111/j.1467-8624.2010.01564.x, PMID: 21291449

[ref16] Eagly A. H. Wood W. Diekman A. B. (2000). “Social role theory of sex differences and similarities: a current appraisal” in The Developmental Social Psychology of Gender. ed. Thomas Eckes H. M. T. (Mahwah, NJ, US: Lawrence Erlbaum Associates Publishers), 123–174.

[ref17] Erath S. A. Elsheikh M. Cummings E. M. (2009). Harsh parenting and child externalizing behavior: skin conductance level reactivity as a moderator. Child Dev. 80, 578–592. doi: 10.1111/j.1467-8624.2009.01280.x, PMID: 19467012 PMC2881831

[ref18] Ford J. S. (2007). Conservation of resources theory. Stress Cult. Community 44, 51–87. doi: 10.1007/978-1-4899-0115-6_3

[ref19] Gershoff E. T. Grogan-Kaylor A. Lansford J. E. Chang L. Zelli A. Deater-Deckard K.. (2010). Parent discipline practices in an international sample: associations with child behaviors and moderation by perceived normativeness. Child Dev. 81, 487–502. doi: 10.1111/j.1467-8624.2009.01409.x, PMID: 20438455 PMC2888480

[ref20] Goodman S. H. Gotlib I. H. (1999). Risk for psychopathology in the children of depressed mothers: a developmental model for understanding mechanisms of transmission. Psychol. Rev. 106, 458–490. doi: 10.1037/0033-295x.106.3.458, PMID: 10467895

[ref21] Hansotte L. Nguyen N. Roskam I. Stinglhamber F. Mikolajczak M. (2021). Are all burned out parents neglectful and violent? A latent profile analysis. J. Child Fam. Stud. 30, 158–168. doi: 10.1007/s10826-020-01850-x

[ref22] He Y. M. Liu T. Chen Y. W.. (2017). *Influence of Parental Rearing Patterns on Academic Burnout: The Mediating Role of Psychological Capital and Self-control*. Singapore: Paper Presented at the 2017 IEEE International Conference on Industrial Engineering and Engineering Management (IEEM).

[ref23] Huang H. M. (2006). Do print and web surveys provide the same results? Comput. Hum. Behav. 22, 334–350. doi: 10.1016/j.chb.2004.09.012

[ref24] Huang J. P. Xia W. Sun C. H. Zhang H. Y. Wu L. J. (2009). Psychological distress and its correlates in Chinese adolescents. Aust. N. Z. J. Psychiatry 43, 674–681. doi: 10.1080/00048670902970817 19530025

[ref25] Kantor G. K. Holt M. K. Mebert C. J. Straus M. A. Drach K. M. Ricci L. R.. (2004). Development and preliminary psychometric properties of the multidimensional neglectful behavior scale-child report. Child Maltreat. 9, 409–428. doi: 10.1177/1077559504269530, PMID: 15538039

[ref26] Kendler K. S. Gardner C. O.. (2017). Genetic and Environmental Influences on Last-year Major Depression in Adulthood: A Highly Heritable Stable Liability but Strong Environmental Effects on 1-Year Prevalence 47. United Kingdom: Cambridge University Press. 10.1017/S0033291717000277 28196550

[ref27] Kessler R. C. Andrews G. Colpe L. J. Hiripi E. Mroczek D. K. Normand S. L. T.. (2002). Short screening scales to monitor population prevalences and trends in non-specific psychological distress. Psychol. Med. 32, 959–976. doi: 10.1017/S0033291702006074, PMID: 12214795

[ref28] Krabbe D. Ellbin S. Nilsson M. Jonsdottir I. R. H. Samuelsson H. (2017). Executive function and attention in patients with stress-related exhaustion: perceived fatigue and effect of distraction. Stress 20, 333–340. doi: 10.1080/10253890.2017.1336533, PMID: 28554267

[ref29] Lay-Khim G. Bit-Lian Y. (2019). Simulated patients' experience towards simulated patient-based simulation session: a qualitative study. Sci. Med. J. 1, 55–63. doi: 10.28991/SciMedJ-2019-0102-3

[ref30] Ling L. Qin S. Shen L. F. (2014). An investigation about learning burnout in medical college students and its influencing factors. Int. J. Nurs. Sci. 1, 117–120. doi: 10.1016/j.ijnss.2014.02.005

[ref31] Liu L. Zhai P. Wang M. (2022). Parental harsh discipline and migrant children's anxiety in China: the moderating role of parental warmth and gender. J. Interpers. Violence 37, NP18761–NP18783. doi: 10.1177/08862605211037580, PMID: 34399600

[ref32] Luo Y. Chen A. Wang Z. (2016). The relationship between parenting style and middle school Students' academic burnout: the mediating role of S elf-concept. Psychol. Dev. Educ. 32, 65–72. doi: 10.16187/j.cnki.issn1001-4918.2016.01.09

[ref33] Maslach C. Jackson S. E. (1981). The measurement of experienced burnout. J. Organ. Behav. 2, 99–113. doi: 10.1002/job.4030020205

[ref34] Mikolajczak M. Brianda M. E. Avalosse H. Roskam I. (2018a). Consequences of parental burnout: its specific effect on child neglect and violence. Child Abuse Negl. 80, 134–145. doi: 10.1016/j.chiabu.2018.03.025, PMID: 29604504

[ref35] Mikolajczak M. Gross J. J. Roskam I. (2019). Parental burnout: what is it, and why does it matter? Clin. Psychol. Sci. 7, 1319–1329. doi: 10.1177/2167702619858430

[ref36] Mikolajczak M. Raes M. E. Avalosse H. Roskam I. (2018b). Exhausted parents: sociodemographic, child-related, parent-related, parenting and family-functioning correlates of parental burnout. J. Child Fam. Stud. 27, 602–614. doi: 10.1007/s10826-017-0892-4

[ref37] Mikolajczak M. Roskam I. (2018). A theoretical and clinical framework for parental burnout: the balance between risks and resources (BR2). Front. Psychol. 9:886. doi: 10.3389/fpsyg.2018.00886, PMID: 29946278 PMC6006266

[ref38] Moons W. G. Eisenberger N. I. Taylor S. E. (2010). Anger and fear responses to stress have different biological profiles. Brain Behav. Immun. 24, 215–219. doi: 10.1016/j.bbi.2009.08.009, PMID: 19732822

[ref39] Ogbueghu S. Aroh P. Robert A. Dauda J. Yahaya J. Nwefuru B.. (2019). Gender differences in academic burnout among economics education students. Global J. Health Sci. 11, 52–57. doi: 10.5539/gjhs.v11n14p52

[ref40] Ridner S. H. (2004). Psychological distress: concept analysis. J. Adv. Nurs. 45, 536–545. doi: 10.1046/j.1365-2648.2003.02938.x 15009358

[ref41] Rogers W. M. Schmitt N. (2004). Parameter recovery and model fit using multidimensional composites: a comparison of four empirical parceling algorithms. Multivar. Behav. Res. 39, 379–412. doi: 10.1207/S15327906MBR3903_1

[ref42] Rohner R. P. (2004). The parental "acceptance-rejection syndrome": universal correlates of perceived rejection. Am. Psychol. 59, 830–840. doi: 10.1037/0003-066X.59.8.830, PMID: 15554863

[ref43] Roskam I. Aguiar J. Akgun E. Arikan G. City M. (2021). Parental burnout around the globe: a 42-country study international investigation of parental burnout. Affect. Sci. 2, 58–79. doi: 10.1007/s42761-020-00028-4, PMID: 33758826 PMC7970748

[ref44] Roskam I. Brianda M.-E. Mikolajczak M. (2018). A step forward in the conceptualization and measurement of parental burnout: the parental burnout assessment (PBA). Front. Psychol. 9:758. doi: 10.3389/fpsyg.2018.00758, PMID: 29928239 PMC5998056

[ref45] Roskam I. Mikolajczak M. (2020). Gender differences in the nature, antecedents and consequences of parental burnout. Sex Roles J. Res. 83, 485–498. doi: 10.1007/s11199-020-01121-5

[ref46] Roskam I. Raes M. E. Mikolajczak M. (2017). Exhausted parents: development and preliminary validation of the parental burnout inventory. Front. Psychol. 8:163. doi: 10.3389/fpsyg.2017.00163, PMID: 28232811 PMC5298986

[ref47] Rudolph K. D. Lambert S. F. Clark A. G. Kurlakowsky K. D. (2001). Negotiating the transition to middle school: the role of self-regulatory processes. Child Dev. 72, 929–946. doi: 10.1111/1467-8624.00325, PMID: 11405592

[ref48] Salmela-Aro K. Savolainen H. Holopainen L. (2009). Depressive symptoms and school burnout during adolescence: evidence from two cross-lagged longitudinal studies. J. Youth Adolesc. 38, 1316–1327. doi: 10.1007/s10964-008-9334-3, PMID: 19779808

[ref49] Smith K. J. Emerson D. J. (2021). Resilience, psychological distress, and academic burnout among accounting students. Acc. Perspect. 20, 227–254. doi: 10.1111/1911-3838.12254

[ref50] Straus M. A. Hamby S. L. Finkelhor D. Moore D. W. Runyan D. (1998). Identification of child maltreatment with the parent–child conflict tactics scales: development and psychometric data for a national sample of American parents. Child Abuse Negl. 22, 249–270. doi: 10.1016/S0145-2134(97)00174-9, PMID: 9589178

[ref51] Wang M. Liu L. (2014). Parental harsh discipline in mainland China: prevalence, frequency, and coexistence. Child Abuse Negl. 38, 1128–1137. doi: 10.1016/j.chiabu.2014.02.016, PMID: 24661692

[ref52] Wang M. Liu L. (2018). Reciprocal relations between harsh discipline and children's externalizing behavior in China: a 5-year longitudinal study. Child Dev. 89, 174–187. doi: 10.1111/cdev.12724, PMID: 28146336

[ref53] Wang M. Wang X. Liu L. (2016). Paternal and maternal psychological and physical aggression and children's anxiety in China. Child Abuse Negl. 51, 12–20. doi: 10.1016/j.chiabu.2015.11.018, PMID: 26704300

[ref54] Wu Y. Dai X. Y. Zhang J. (2007). Preliminary development of learning burnout questionnaire for junior high school students. Chin. J. Clin. Psych. 15:764824, 118–121. doi: 10.3969/j.issn.1005-3611.2007.02.003

[ref55] Wu K. Wang F. Wang W. Li Y. (2022). Parents’ education anxiety and Children’s academic burnout: the role of parental burnout and family function. Front. Psychol. 12:764824. doi: 10.3389/fpsyg.2021.764824, PMID: 35185673 PMC8855929

[ref56] Xie X. Chen W. Zhu X. He D. (2019). Parents' phubbing increases Adolescents' Mobile phone addiction: roles of parent-child attachment, deviant peers, and gender. Child Youth Serv. Rev. 105:104426. doi: 10.1016/j.childyouth.2019.104426

[ref57] Yang B. Chen B. B. Qu Y. Zhu Y. (2021). Impacts of parental burnout on Chinese Youth's mental health: the role of Parents' autonomy support and emotion regulation. J. Youth Adolesc. 50, 1679–1692. doi: 10.1007/s10964-021-01450-y, PMID: 34106359 PMC8188764

[ref58] Yang H. J. Cheng K. F. (2005). An investigation the factors affecting MIS student burnout in technical-vocational college. Comput. Hum. Behav. 21, 917–932. doi: 10.1016/j.chb.2004.03.001

[ref59] Zhang H. Gao T. Hu Q. Zhao L. Wang X. Sun X.. (2022). Parental marital conflict, negative emotions, phubbing, and academic burnout among college students in the postpandemic era: a multiple mediating models. Psychol. Sch. 1:22707. doi: 10.1002/pits.22707

